# Plasmonic Waveguide Modulator Integrated with a Transferred Bi_2_Se_3_ Topological Insulator Film

**DOI:** 10.1021/acsomega.6c04951

**Published:** 2026-08-03

**Authors:** Cheng-Yi Cheng, Kuan-Han Wu, Jiang-Qin Shi, Rohit Gupta, Jian-Jang Huang

**Affiliations:** † Graduate Institute of Photonics and Optoelectronics, 33561 National Taiwan University, No. 1, Sec. 4, Roosevelt Rd., Taipei 10639, Taiwan; ‡ Department of Electrical Engineering, National Taiwan University, No. 1, Sec. 4, Roosevelt Rd., Taipei 10639, Taiwan

## Abstract

We demonstrate a voltage-tunable plasmonic modulator that integrates a Bi_2_Se_3_ topological insulator thin film with a metal–dielectric plasmonic waveguide. The Bi_2_Se_3_ layer, transferred using a polymer-assisted method, enhances the modulation depth of the waveguide by enabling electrically driven carrier redistribution at the Au/Bi_2_Se_3_ interface, which perturbs the effective dielectric response of the shared Au plasmonic layer. With the Bi_2_Se_3_ film, the device demonstrates a significant output peak displacement in the transmission spectrum of up to 49.2 nm, along with an enhanced extinction ratio (ER) of 4.21 dB under an applied bias of 10 V for a 100 μm-long waveguide. These modulation effects are attributed to Au/Bi_2_Se_3_ interfacial carrier redistribution, carrier-induced modification of the shared Au plasmonic layer, possible Burstein–Moss-related absorption modification in Bi_2_Se_3_, and accumulated wavelength-dependent propagation loss. Our results suggest that the unique properties of Bi_2_Se_3_ offer a viable approach for broadband optical modulation, providing an alternative to conventional 2D materials like graphene. This work highlights the utility of topological materials for developing actively tunable components in integrated nanophotonic systems.

## Graphical abstract

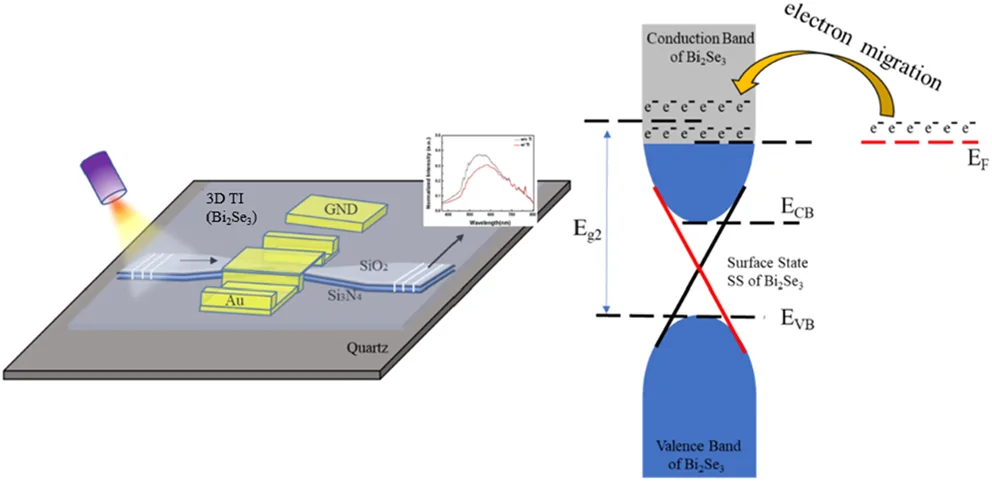

## Introduction

Photonic devices are a compelling alternative to conventional electronic interconnects in high-performance computing systems, offering significantly enhanced bandwidth, energy efficiency, and data transmission speed.^1,2^ The accelerating demands of high-throughput computing platforms have driven the development of copackaged optical systems with increased data rates and more compact integrated circuitry.^3^ Nevertheless, the miniaturization of photonic components is fundamentally constrained by the diffraction limit, which restricts light confinement to approximately half of its transmission wavelength.^4,5^ Plasmonic waveguides have garnered considerable attention to overcome these limitations, enabling the manipulation and confinement of electromagnetic waves at subwavelength scales and facilitating optical signal transmission beyond the diffraction limit.^5–7^

Plasmonic waveguides operate by coupling light with collective electron oscillations, surface plasmons, at metal–dielectric interfaces, allowing for subwavelength confinement of optical modes. The primary classifications of plasmonic waveguides include metal–insulator–metal (MIM),^8,9^ insulator–metal–insulator (IMI),^10,11^ and hybrid plasmonic waveguides.^12,13^ MIM waveguides offer exceptional mode confinement due to the tight localization of the optical field within the dielectric gap, thus enabling ultracompact photonic circuitry. However, they are inherently limited by high propagation losses from strong absorption in the metallic layers. In contrast, IMI waveguides exhibit reduced confinement but benefit from lower optical losses, making them favorable for applications requiring extended propagation lengths. Hybrid plasmonic waveguides seek a balance by combining a high-confinement metal structure with a low-loss dielectric, thereby optimizing the trade-off between propagation loss and field confinement. The choice of waveguide architecture thus depends critically on the specific application requirements, such as integration density, propagation length, or modulation depth.

Recently, a combination of plasmonic waveguides with two-dimensional (2D) materials such as graphene has been demonstrated for modulation.^14–16^ Graphene is considered a promising candidate for next-generation photonic modulators due to its exceptional properties, including high thermal conductivity, electrical current capacity, and carrier mobility. However, the small graphene thickness implies that only a limited percentage of light can be absorbed or modulated by the gating voltage. The modulation depth is thus limited to ∼0.1 dB per graphene layer.^14^

To address these limitations while leveraging the advantageous electronic properties of 2D or thin film materials, we propose a novel hybrid plasmonic modulator incorporating a metal–dielectric waveguide structure coated with a topological insulator (TI) thin film. The carrier numbers in the TI, with the insulating bulk and conducting surface, can be readily modulated by the applied electric field. We choose Bi_2_Se_3_ as the thin film on the plasmonic waveguide because Bi_2_Se_3_ is a 3D TI film with conductive surface states with high mobility^17^ at room temperature. We adopt the Burstein–Moss effect^18^ to manipulate carrier injection and depletion between TI and metal. Given the challenges associated with the direct growth of high-quality Bi_2_Se_3_ films on prefabricated plasmonic structures, we develop a polymer-assisted transfer technique that facilitates the integration of Bi_2_Se_3_ thin films onto the waveguide surface without compromising film quality. Using this approach, we fabricate and characterize a voltage-tunable plasmonic modulator, systematically benchmarking its performance and optical modulation behavior in the presence of the transferred Bi_2_Se_3_ film.

## Experimental Methods

### Waveguide Fabrication and Measurement Setup

The fabrication process of the plasmonic waveguide is illustrated in Figure 1a. We adopted a hybrid MIS (metal–insulator-semiconductor) plasmonic waveguide structure. The waveguide arrangement results in a plasmonic peak at the wavelength of around 540 nm.^19,20^ The process begins with depositing an 180 nm-thick silicon nitride (Si_3_N_4_) layer onto a quartz substrate, followed by a 20 nm-thick silicon dioxide (SiO_2_) layer atop the Si_3_N_4_. The waveguide structure is subsequently defined using reactive ion etching (RIE), yielding a width of 4.5 μm and variable lengths. A 220 nm-thick gold (Au) layer is then deposited via electron-beam evaporation to serve as the plasmonic layer. An additional 350 nm-thick Au layer is deposited as probe pads that serve as a bias pad and a ground pad on both sides along the waveguide to facilitate electrical probing. Light propagates along the high-refractive-index Si_3_N_4_ waveguide in this configuration, while surface plasmon polaritons (SPPs) are excited at the Au/SiO_2_ interface. A grating coupler with a period of 6 μm is then defined by depositing a 100 nm-thick Si_3_N_4_ layer, followed by patterning via RIE, to facilitate efficient coupling of free-space light into the waveguide. For a device without subsequent Bi_2_Se_3_ thin film transfer, VIA holes are opened by RIE for contact probing. As for the plasmonic waveguide designed for Bi_2_Se_3_ film coating, we next transfer a Bi_2_Se_3_ thin film of 30 nm thickness using a polymer-assisted method to introduce active modulation functionality. Finally, a 350 nm-thick Au layer is deposited on top of the Bi_2_Se_3_ film along both sides of the waveguide to serve as the top electrode, thereby completing the device fabrication. An optical microscopic image of the fabricated plasmonic waveguide is presented in Figure 1a. The experimental setup for optical and electrical characterization is shown in Figure 1b. A broadband visible light source with a 300–700 nm spectral range is employed for optical measurements. Light is coupled into the waveguide using an optical fiber, a linear polarizer, a focal lens, and the fabricated grating coupler. Since the surface plasmon polaritons (SPPs) at the Au/SiO_2_ interface are contributed from the TM mode of light within the waveguide, in this work, TM-polarized light is selected before coupling to the waveguide. The output light is collected at the waveguide terminus via another optical fiber and analyzed using a spectrometer. Simultaneously, electrical measurements are conducted using a Keysight 4155C Semiconductor Parameter Analyzer. A bias voltage ranging from 0 V to 10 V is applied across the contact electrodes, with precise voltage control achieved through a computer interface.

**Figure 1:**
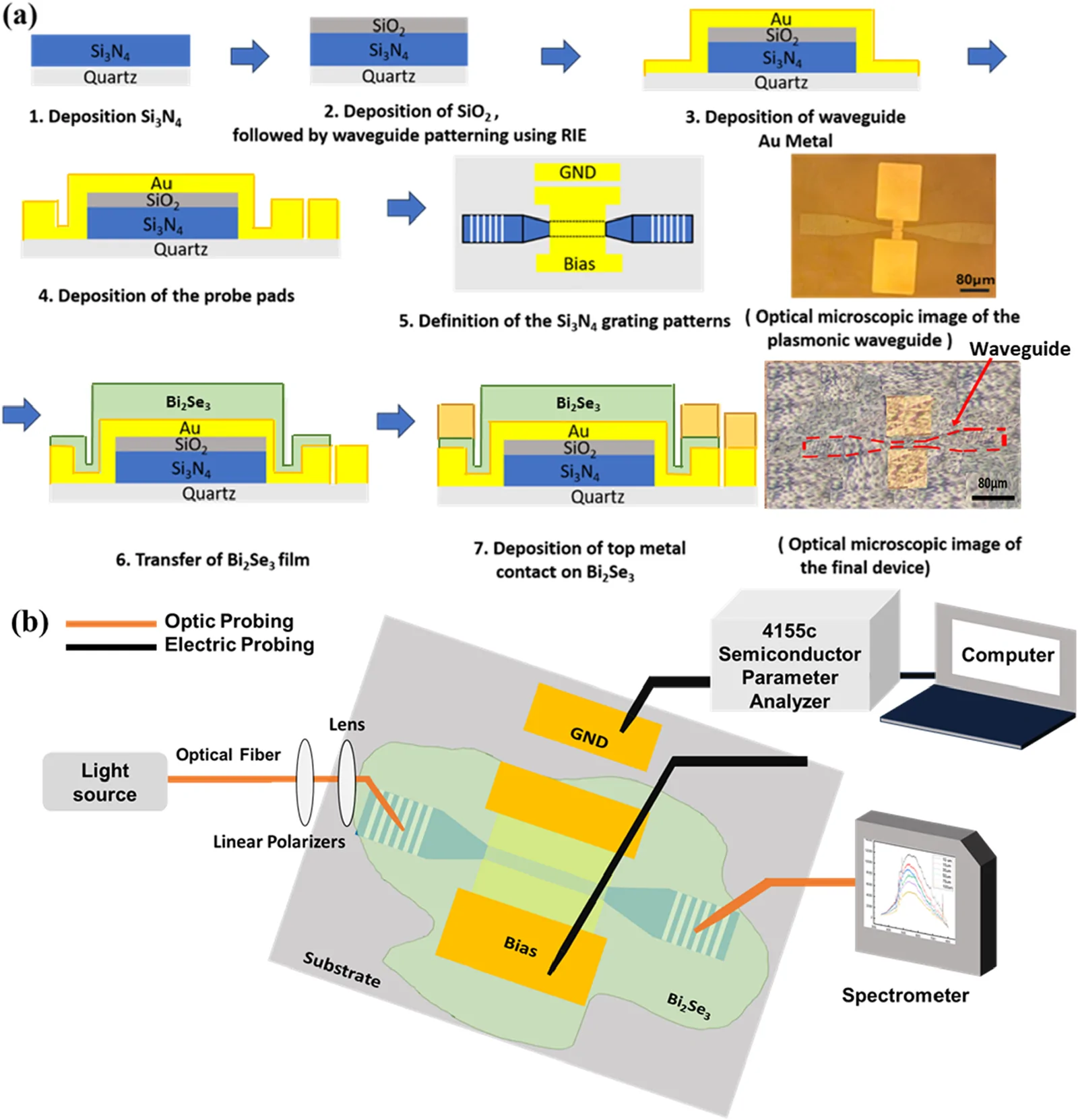
(a) Process flow of the plasmonic waveguide fabrication, including the deposition of the Si_3_N_4_ and SiO_2_ dielectric, waveguide patterning, Au film deposition, and grating formation. Once the plasmonic waveguide is fabricated, the Bi_2_Se_3_ is transferred onto the device, and the metal pads for bias on both sides of the waveguide are deposited. (For the Bias means, the pad where we induced bias, and GND is for the ground pad.) (b) Experimental setup for characterizing the plasmonic waveguide. (light source for Thorlabs SLS205, 75 W, wavelength 300–700 nm, optical fiber diameter 105 ± 2.1 μm, lens for Thorlabs LA1951-A, focus = 25 mm).

### Synthesis and Transfer of the Bi_2_Se_3_ Thin Film

The Bi_2_Se_3_ thin films were synthesized using a custom-built vapor phase deposition system, as illustrated in Figure 2. The process begins with purging the furnace chamber with high-purity argon gas at room temperature to eliminate residual contaminants. Subsequently, 600 mg of Bi_2_Se_3_ powder is placed in the source zone (Zone B) of the furnace (Figure 2a). Meanwhile, the bare sapphire (Al_2_O_3_(0001)) substrates with a size of 1 × 1 cm^2^, precleaned using an Ar gun, and the samples are placed on Zone A and C in the furnace. The furnace is then gradually heated over 50 min until the temperatures of the three zones reach 300 °C (upstream), 800 °C (center), and 300 °C (downstream), respectively. The deposition process is maintained for 180 min to facilitate the growth of uniform thin films. An in situ annealing process is employed immediately following deposition to minimize surface defects and achieve a smoother film morphology. Specifically, the central zone temperature is rapidly quenched to 20 °C, while the outer zones are simultaneously ramped from 300 to 650 °C. This thermal gradient promotes effective postdeposition annealing, enhancing surface quality. After annealing, the furnace is cooled naturally to room temperature.^21,22^ The temperature profiles in the furnace are shown in Figure 2b. The material properties of the Bi_2_Se_3_ thin film synthesized using the above steps can be referred to our earlier work in ref^23^. Due to challenges associated with directly depositing high-quality Bi_2_Se_3_ films onto prefabricated plasmonic waveguides using the above synthesis method, we developed a film transfer technique to enable integration of Bi_2_Se_3_ onto the waveguide structure.

**Figure 2:**
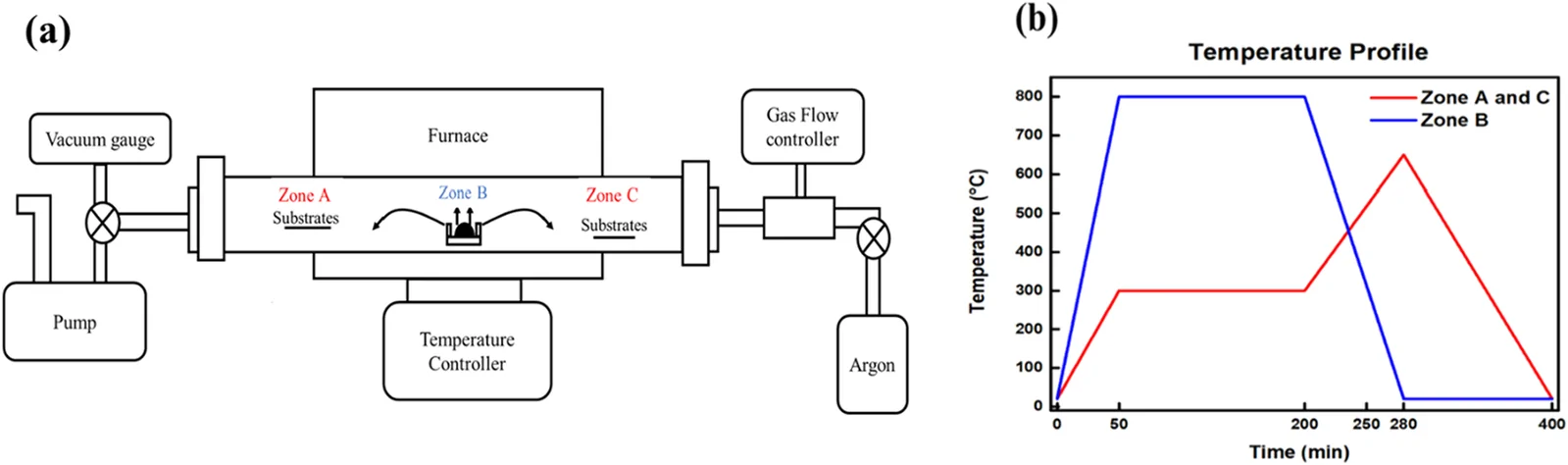
(a) Schematic diagram of the furnace system for Bi_2_Se_3_ growth. (b) Temperature profiles during Bi_2_Se_3_ growth.

As shown in Figure 3a,b, a Bi_2_Se_3_ thin film was deposited on an Al_2_O_3_ (0001) substrate. To facilitate film transfer, poly­(methyl methacrylate) (PMMA 950) was used as a mechanical support layer due to its excellent elasticity and chemical stability in alkaline environments. As illustrated in Figure 3c, a single layer of PMMA was spin-coated onto the as-grown Bi_2_Se_3_ film and baked at 110 °C for 2 min 42 s, ensuring firm adhesion between PMMA and the Bi_2_Se_3_ surface.^24,25^ To initiate delamination, the PMMA-coated Bi_2_Se_3_/Al_2_O_3_ sample was immersed in a potassium hydroxide (KOH) and deionized water mixture heated to 90 °C, which selectively promotes interfacial etching at the Bi_2_Se_3_/Al_2_O_3_ boundary. The PMMA layer remains intact during etching, serving as a flexible support that prevents mechanical cracking of the Bi_2_Se_3_ film. Once the etching front reached the interface, the corners of the Bi_2_Se_3_ film began to lift, driven by wet etching and interfacial stress release (Figure 3d). During this stage, the sample was repeatedly moved between the KOH and deionized water baths to remove byproducts and accelerate the delamination process. After complete separation, the floating PMMA/Bi_2_Se_3_ bilayer was retrieved using the target plasmonic waveguide substrate from beneath the liquid surface (Figure 3e). The transferred film was then heated at 100 °C for 30 min on a hot plate to remove moisture and enhance interfacial adhesion. The sample was stored overnight in a sealed container to stabilize the film, followed by PMMA removal in acetone, resulting in a clean and uniform Bi_2_Se_3_-coated plasmonic waveguide (Figure 3f).

**Figure 3:**
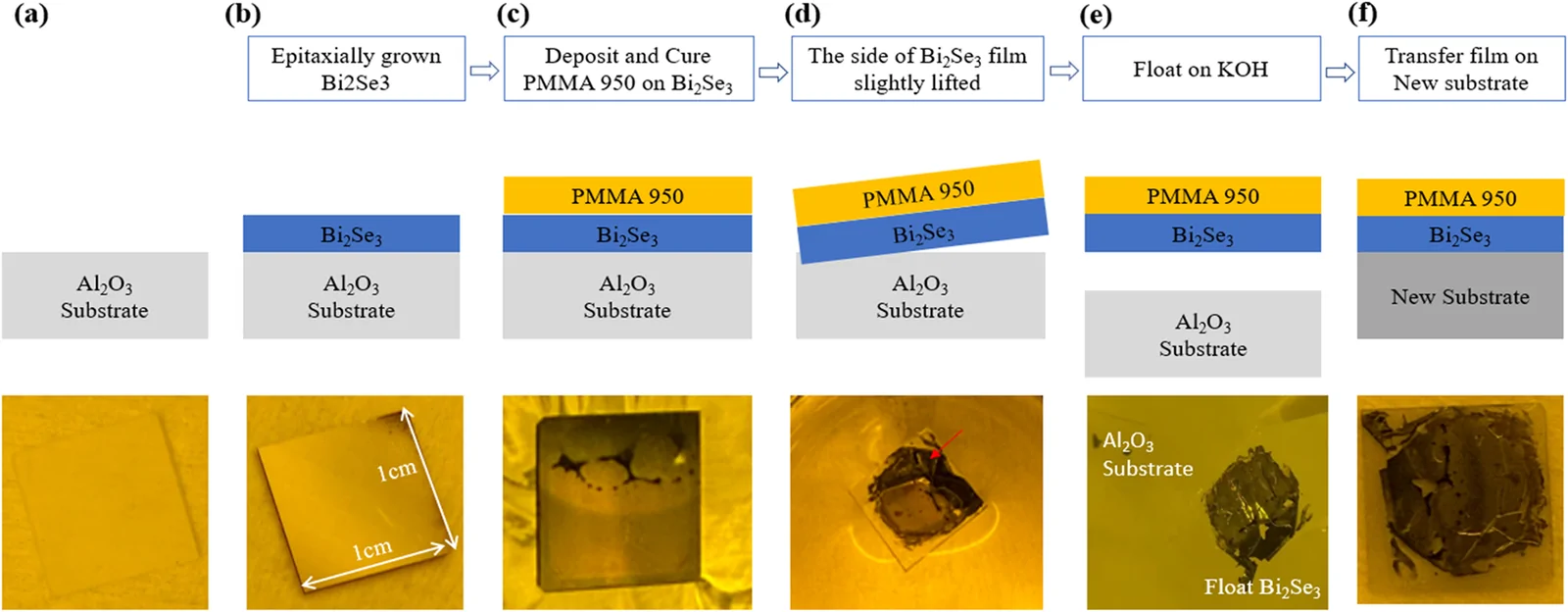
Bi_2_Se_3_ thin film transfer procedure: (a) Substrate cleaning. (b) Bi_2_Se_3_ thin film deposition in the furnace. (c) PMMA 950 layer spin-coating and curing; the sample is then immersed in KOH solution to initiate delamination. (d) Partial delamination of the Bi_2_Se_3_ film. (e) Floating of the PMMA-supported Bi_2_Se_3_ film in the KOH solution. (f) Transfer of the freestanding film to a new substrate.

### X-ray Diffraction (XRD) Analysis

We conducted X-ray diffraction (XRD) analysis to assess the crystallinity and phase purity of the Bi_2_Se_3_ thin films before and after the transfer process. As shown in Figure 4a, the XRD pattern of the as-grown Bi_2_Se_3_ film on an Al_2_O_3_ (0001) substrate exhibits a series of sharp diffraction peaks corresponding to the (003), (006), (009), (0012), (0015), (0018), and (0021) planes of rhombohedral Bi_2_Se_3_, indicating a high degree of *c*-axis orientation.^26,27^ Following the transfer of the Bi_2_Se_3_ film onto a foreign Al_2_O_3_ substrate, the XRD pattern (Figure 4b) reveals the same set of (00l) reflections, though with the reduction in peak intensity. This reduction is likely due to changes in substrate background and minor alterations in film morphology during the transfer. Nevertheless, retaining well-defined (00l) peaks confirms that the transferred film preserves its crystallographic orientation and phase purity. These results collectively demonstrate that the polymer-assisted transfer process does not significantly degrade the crystalline quality or disrupt the out-of-plane texture of the Bi_2_Se_3_ thin film, thereby validating the structural integrity of the material post-transfer.

**Figure 4:**
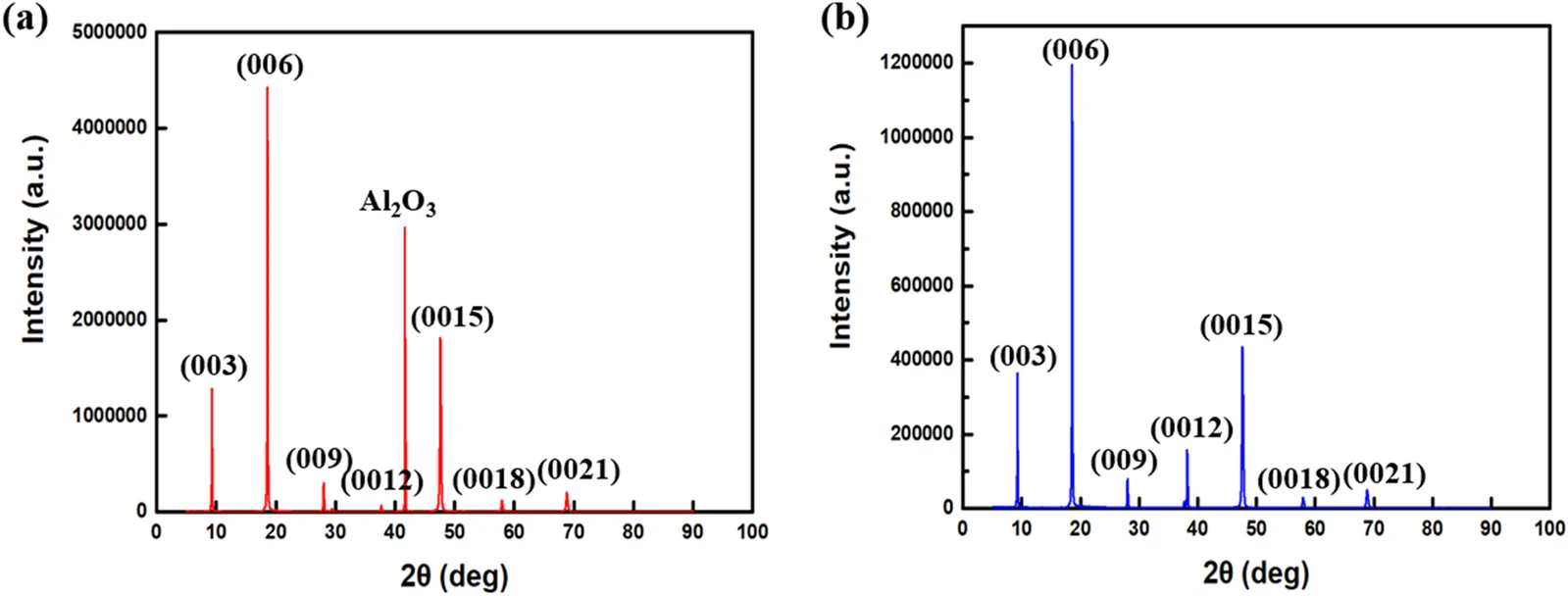
(a) XRD spectrum of the as-grown Bi_2_Se_3_ film on the Al_2_O_3_(0001) substrate. (b) XRD spectrum of the Bi_2_Se_3_ film after transferring to our waveguide device.

## Results and Discussion

### Mode Analysis of the Plasmonic Waveguide with and without Bi_2_Se_3_

Finite-element mode simulations were performed for the plasmonic waveguide structures before and after Bi_2_Se_3_ integration to examine the actual modal confinement and the role of the transferred Bi_2_Se_3_ film. As shown in Figure 5a, the optical field of the reference waveguide without Bi_2_Se_3_ is mainly confined near the Au/SiO_2_ interface, confirming the excitation of the guided SPP mode. After the Bi_2_Se_3_ transfer, the simulated field profile in Figure 5b shows that the dominant SPP field still remains localized at the Au/SiO_2_ interface, with negligible direct optical field overlap with the top Bi_2_Se_3_ layer due to the metallic screening of the 220 nm-thick Au layer. Therefore, the Bi_2_Se_3_ film is considered to influence the plasmonic response mainly through interfacial carrier redistribution and the modification of the effective dielectric boundary condition of the shared Au plasmonic layer that will be discussed later, rather than through direct evanescent-field penetration into the Bi_2_Se_3_ film.

**Figure 5:**
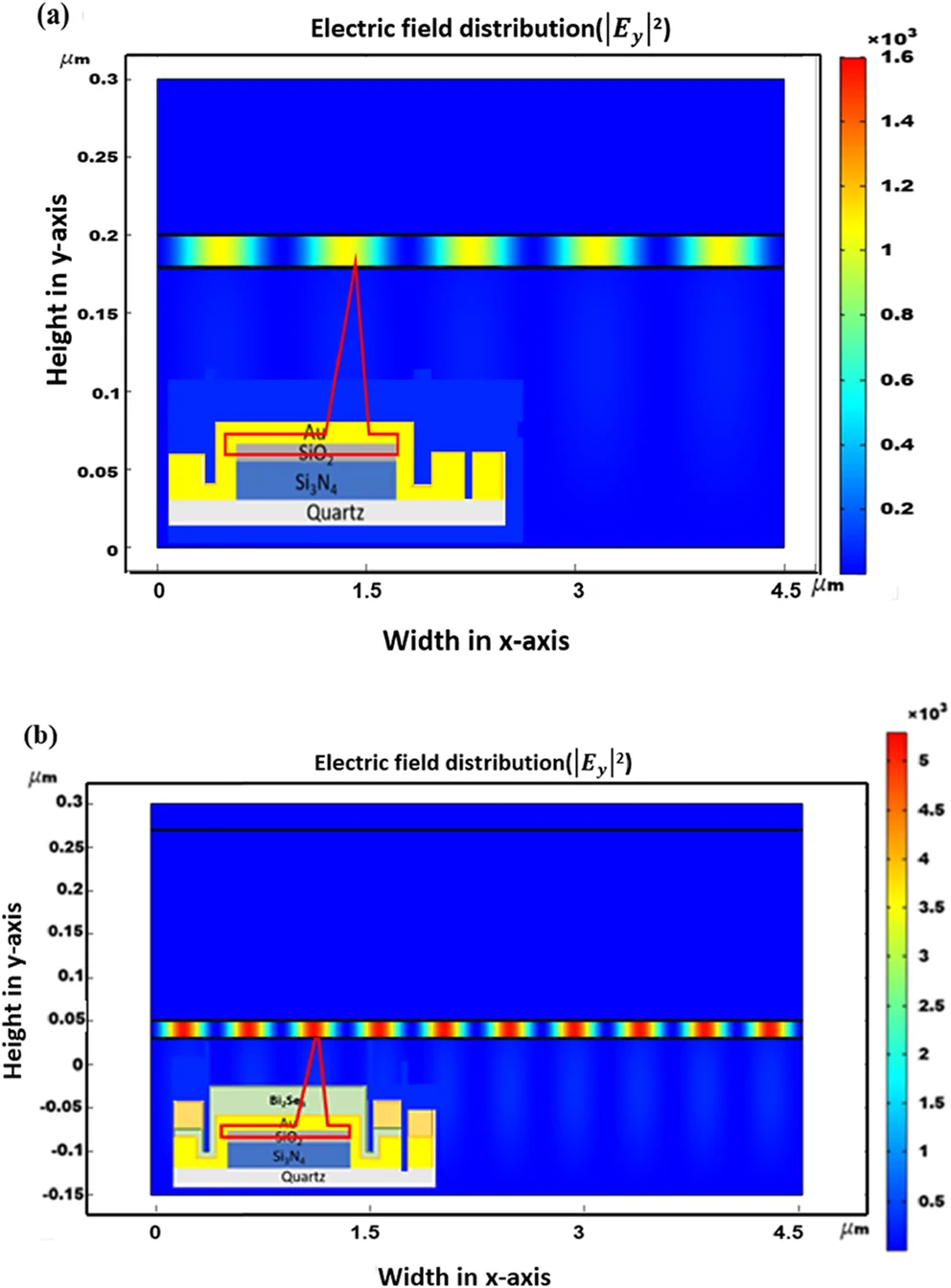
Simulated cross-sectional electric-field distributions of the plasmonic waveguide. (a) Reference waveguide without Bi_2_Se_3_ and (b) Bi_2_Se_3_-integrated waveguide. The dominant optical field is localized near the Au/SiO_2_ interface in both structures, confirming the guided SPP mode supported by the Au/SiO_2_ boundary. After the Bi_2_Se_3_ transfer, no significant direct optical-field overlap with the top Bi_2_Se_3_ layer is observed due to the screening effect of the 220 nm-thick Au layer.

### Voltage-Controlled Plasmonic Responses in the Waveguide without Bi_2_Se_3_

Because SPPs arise from the TM polarization of light, the plasmonic responses of the modulator were benchmarked by coupling TM-polarized light to the waveguide. The Supporting Information provides a comparison of the modulation behaviors for TM mode and TE mode light incidence. This comparison indicates that no obvious modulation is observed with the TE mode incident light. The TM-mode-dependent modulation behavior clearly demonstrates the plasmonic properties of our waveguide.

The normalized transmission spectra (TM polarization) measured at the output of plasmonic waveguides with varying lengths and the input light source spectrum are presented in Figure 6a. The spectra are normalized to the emission profile of the broadband light source (see the inlet of Figure 6a) to highlight wavelength-dependent transmission behavior. Although SPPs are theoretically supported at the Au/SiO_2_ interface over a broad spectral range of 400–800 nm,^28,29^ the experimental results reveal a distinct transmission band centered within the 500–650 nm range. This spectral window corresponds to the efficient coupling regime, where the guided optical mode strongly interacts with the metal interface via SPP excitation. As the waveguide length increases, the overall transmission intensity decreases, which can be attributed to the cumulative effects of absorption and scattering losses along the propagation path.^29^ These losses primarily arise from the interaction of the evanescent field with the inherently lossy metal interface. Moreover, the observed peak wavelength exhibits a slight output peak displacement with increasing waveguide length, indicating that geometric parameters influence the effective refractive index and the SPP coupling condition. This output peak displacement is consistent with increased optical path length and modified modal dispersion characteristics in longer waveguides.

**Figure 6:**
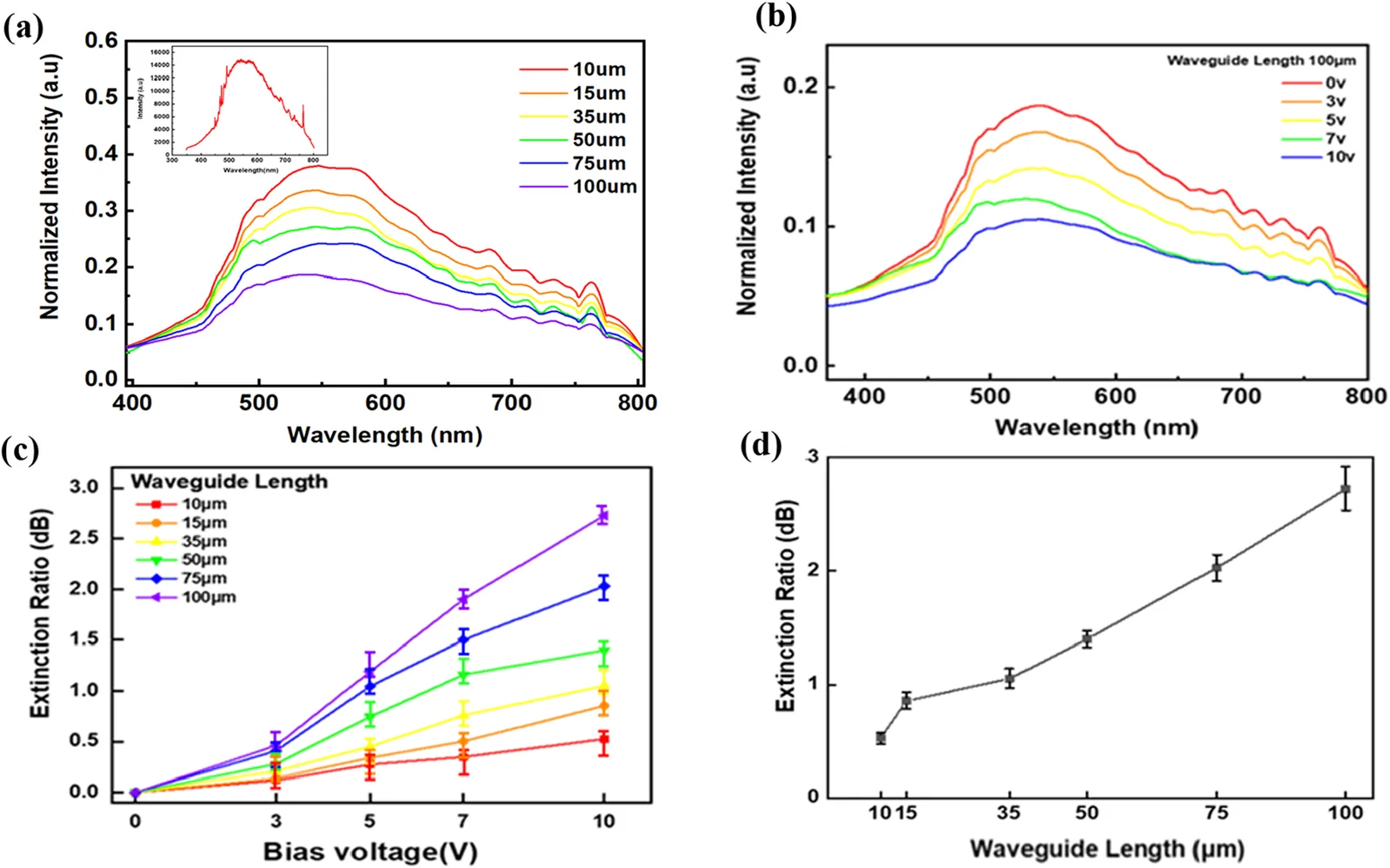
(**a)** Normalized transmission spectra of the plasmonic waveguides with various lengths. No external voltage was applied. Inlet: Input light source spectrum. (b) Normalized transmission spectra of a 100 μm-long waveguide at various applied voltages. (c) ER of the plasmonic waveguides with various lengths, extracted from the peak transmission intensity under each bias condition. (d) ER at a bias voltage of 10 V in waveguides with various lengths. Each data point represents the average value obtained from three independent devices with the same nominal structure, and the error bars indicate one standard deviation.

The normalized transmission spectra exhibit a further decrease upon applying an external bias, as illustrated in Figure 6b using the 100 μm-long waveguide as a representative example. This modulation arises from bias-induced changes in the optical properties of gold and alterations in the modal field distribution at the metal–dielectric interface. The applied electric field on the gold pad increases the real and imaginary parts of the gold’s refractive index.^31^ An increase in the real part modifies the interfacial impedance, leading to greater reflection and reduced coupling efficiency between the guided mode and surface plasmon polaritons (SPPs). Concurrently, an increase in the imaginary component enhances intrinsic optical absorption within the metal, thereby reducing the transmitted optical power.

In addition to modifying Au’s optical constants, the applied bias also induces surface charge accumulation and localized polarization at the Au/SiO_2_ interface.^31,32^ These effects enhance the local electric field intensity, thereby strengthening SPP excitation. The resultant increase in field confinement near the metal surface promotes stronger interaction with the lossy metallic region, ultimately leading to elevated Ohmic losses. Notably, this modulation behavior exhibits a pronounced wavelength dependence, reflecting the dispersive nature of the material response and SPP coupling efficiency under applied bias.

A parameter, the ER, is employed to quantify the modulation performance and is expressed as

1 $$\mathrm{ER}=10\log_{10}I_{\mathrm{off}}-10\log_{10}I_{\mathrm{on}}$$

where *I*_on_ and *I*_off_ are the transmitted peak optical intensities measured with and without the applied voltage, respectively. ER represents the relative transmission change induced by the external modulation.

ER exhibits a clear dependence on the waveguide length. The waveguide length increases from 10 to 100 μm, and the interaction between the guided optical mode and the metallic interface becomes increasingly significant. As shown in Figure 6c, this extended interaction length enhances the efficacy of voltage-induced modulation by increasing the overlap between the optical mode and the plasmonic field. Moreover, Figure 6d summarizes the ER of various waveguide lengths at a bias of 10 V. For a 100 μm waveguide length, the ER is 2.73 dB at the wavelength of 540 nm.

### Plasmonic Responses in the Waveguide with the Transferred Bi_2_Se_3_ Thin Film

We further investigated the modulation of plasmonic wave propagation along the waveguide upon introducing a Bi_2_Se_3_ thin film. As illustrated in Figure 7a, using the 100 μm-long waveguide as a representative example, the normalized transmission spectrum exhibits a noticeable output peak displacement compared to the control device without Bi_2_Se_3_. At 0 V bias, the measured insertion loss of the 100 μm-long reference waveguide without Bi_2_Se_3_ is 45.57 dB, while that of the Bi_2_Se_3_-integrated waveguide increases to 52.57 dB. After subtracting the corresponding coupling losses of 20.54 and 25.14 dB, the propagation losses are estimated to be 0.250 dB/μm and 0.274 dB/μm for the devices without and with Bi_2_Se_3_, respectively. The increased loss after Bi_2_Se_3_ integration is mainly attributed to carrier redistribution at the Au/Bi_2_Se_3_ interface, which modifies the effective dielectric response of the shared Au plasmonic layer and increases the attenuation of the Au/SiO_2_-supported SPP mode, rather than direct optical-field absorption in the Bi_2_Se_3_ film.

**Figure 7:**
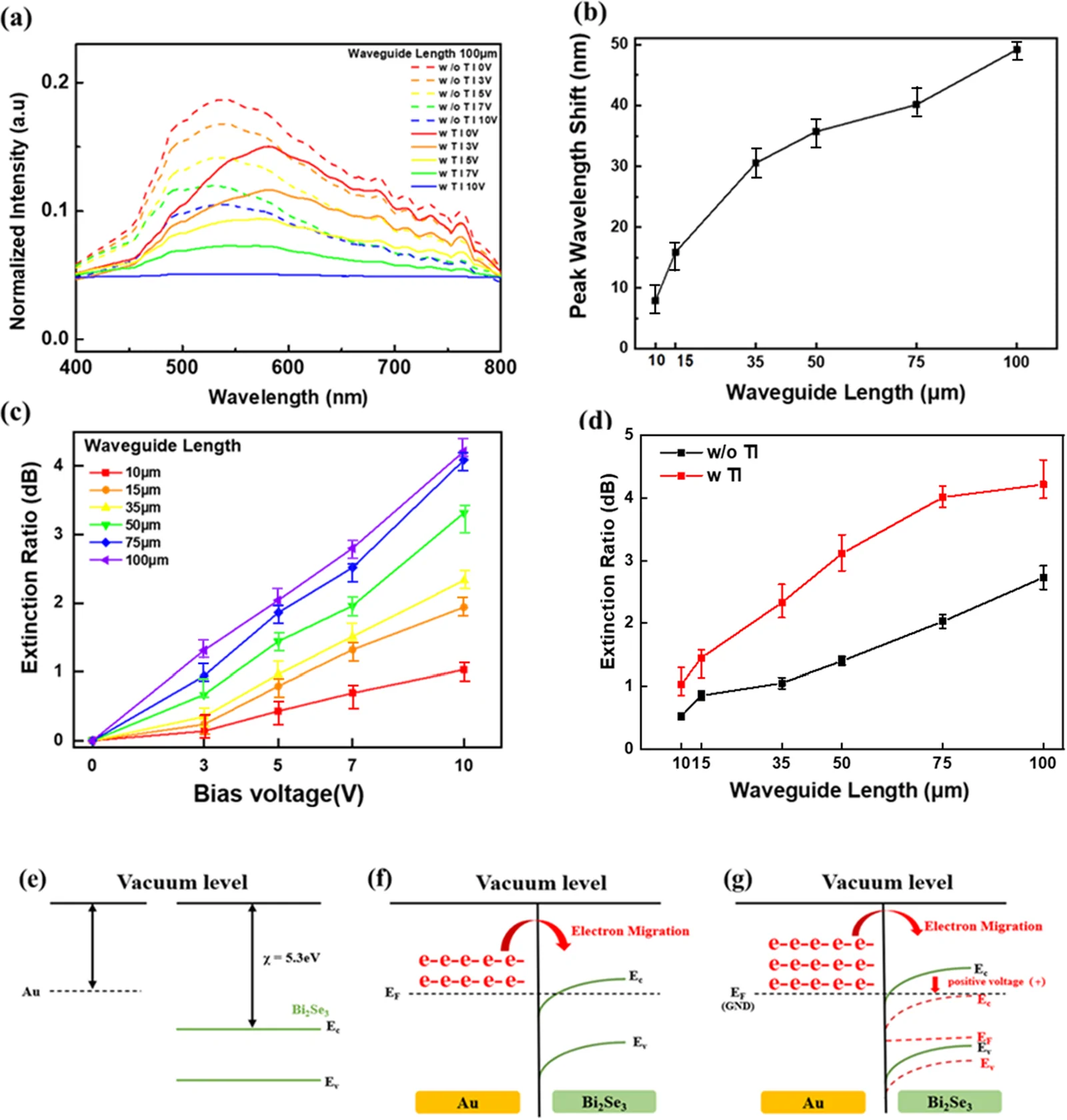
(a) Transmission spectra of a 100 μm-long waveguide with and without coating a Bi_2_Se_3_ film. (b) Output peak displacement of waveguides with different lengths. (c) ER vs bias voltage of different waveguide lengths with Bi_2_Se_3_. (d) Comparisons of ERs with and without Bi_2_Se_3_ at different waveguide lengths. Each data point represents the average value obtained from three independent devices with the same nominal structure, and the error bars indicate one standard deviation. (e) Schematic energy band diagrams of Au and Bi_2_Se_3_ before contact. (f) Schematic diagram of the Fermi-level alignment when Au and Bi_2_Se_3_ contact each other. (g) Band diagram of Au/Bi_2_Se_3_ under positive bias on the Bi_2_Se_3_ layer. Note that the Au in the band diagram refers to the Au layer on the waveguide, instead of the Au contact pad beside the waveguide.

Figure 7b summarizes the output peak displacement with various waveguide lengths. As the waveguide length increases from 10 to 100 μm, the peak displacement increases from 7.9 to 49.2 nm. Here, the Bi_2_Se_3_-induced peak displacement is defined as the difference between the peak wavelength of the Bi_2_Se_3_-integrated device and that of the reference device without Bi_2_Se_3_ under the same waveguide length. Therefore, the measured displacement of 7.9–49.2 nm represents the total additional spectral modification introduced by Bi_2_Se_3_ integration, rather than a separately isolated contribution from each individual mechanism.

The observed length-dependent spectral displacement should be interpreted as an apparent peak shift caused by cumulative wavelength-dependent attenuation during SPP propagation, rather than a direct length-dependent shift of the intrinsic resonance wavelength.^29^ In general, the resonance wavelength determined by the effective refractive index variation is not expected to strongly depend on the physical propagation length. Therefore, the increasing peak displacement with the waveguide length indicates that the output spectrum is progressively reshaped by propagation loss.^30,33^

After Bi_2_Se_3_ integration, the optical response of the hybrid plasmonic channel is modified by the intrinsic absorption and complex refractive index of Bi_2_Se_3_ in the visible–near-infrared region,^34^ as well as by carrier redistribution at the Au/Bi_2_Se_3_ interface.^35^ These effects introduce stronger wavelength-dependent attenuation on the short-wavelength side of the transmission band. As the propagation length increases, this accumulated attenuation progressively suppresses the high-energy side of the transmission spectrum, causing the apparent output peak to move toward longer wavelengths.

The Burstein–Moss effect is also considered as part of this carrier-induced optical modification.^18^ Electron transfer from Au to Bi_2_Se_3_ can change the carrier occupation and absorption characteristics of Bi_2_Se_3_, while the associated Au/Bi_2_Se_3_ interfacial charge redistribution modifies the local complex refractive index and loss of the hybrid plasmonic channel.^35^

When a positive voltage is applied to the gold contact pad above the Bi_2_Se_3_ film, the TI film possesses a higher potential than the bottom Au/SiO_2_ stack that supports the plasmonic mode. As shown in Figure 7c, the ER increases with applied bias (on top gold pad) for all waveguide lengths, indicating a voltage-dependent reduction in transmitted optical power. This trend suggests enhanced optical attenuation due to bias-induced carrier redistribution at the Au/Bi_2_Se_3_ interface, which perturbs the carrier density of the shared Au plasmonic layer supporting the Au/SiO_2_ SPP mode. The resulting change in the carrier population modifies the real and imaginary components of the effective Au-based plasmonic response, thereby increasing absorption loss and reducing the transmitted optical power. These effects collectively lead to a more pronounced attenuation of the transmitted signal under electrical bias.

Furthermore, the observed voltage-dependent modulation is not caused by leakage-current-induced Joule heating. To clarify, the *I*–*V* characteristics of the Bi_2_Se_3_-integrated device were measured under the same 0–10 V bias range. The current remained in the pA range throughout the bias sweep, corresponding to an electrical power dissipation below approximately 200 pW even at 10 V. This low-leakage behavior indicates that Joule heating is negligible, and supports that the observed spectral and intensity modulation mainly originates from electric-field-induced carrier redistribution at the Au/Bi_2_Se_3_ interface rather than thermo-optic tuning.

Figure 7d summarizes the ER as a function of waveguide length. Under an applied voltage of 10 V, the ER increases from 1.03 dB for a 10 μm-long waveguide to 4.21 dB for a 100 μm-long waveguide in devices coated with Bi_2_Se_3_. The increase represents a significant improvement over the uncoated counterparts, where the ER only increases from 0.52 dB to 2.73 dB, as shown in Figure 6d. These results indicate that the Bi_2_Se_3_ layer substantially enhances modulation depth by introducing additional optical loss and enabling voltage-tunable carrier dynamic—effects that are particularly pronounced in longer propagation lengths.

To quantitatively separate the Au-only modulation from the additional Bi_2_Se_3_-induced modulation, the reference device without Bi_2_Se_3_ is used as the control response of the Au-based plasmonic waveguide. At a waveguide length of 100 μm and an applied voltage of 10 V, the reference device exhibits an ER of 2.73 dB, whereas the Bi_2_Se_3_-integrated device reaches 4.21 dB. Therefore, the additional ER introduced by Bi_2_Se_3_ integration is estimated to be 1.48 dB, corresponding to a 54% enhancement compared with the reference device. This additional ER is attributed to the combined effects of TSS-related surface/interface carrier modulation, carrier-induced modification of the shared Au plasmonic layer, and possible Bi_2_Se_3_ bulk-related absorption/Burstein–Moss carrier-filling effects, rather than to a separately isolated contribution from each individual mechanism.^18,34,35^

The energy band diagrams in Figure 7e–g elucidate the underlying carrier transport behavior. As illustrated in Figure 7e, the Fermi level (EF) of Au on the waveguide lies higher than that of Bi_2_Se_3_, which has an electron affinity of ∼5.3 eV.^35^ Upon contact (Figure 7f), electrons are transferred from Au to Bi_2_Se_3_, resulting in Fermi-level alignment and significant downward band bending in Bi_2_Se_3_. This interfacial charge transfer, driven by the high electronegativity of the terminating selenium atoms in Bi_2_Se_3_, accumulates interface charge and pins the Fermi level within the conduction band. Consequently, the conduction band minimum (CBM) is lowered, and the Schottky barrier is effectively eliminated, forming an n-type Ohmic contact even in nominally undoped Bi_2_Se_3_.^25,35^

When a positive bias is applied to the Bi_2_Se_3_ layer (see Figure 1a for the probe contact position and Figure 7g for the band diagram), the resulting electric field partially overcomes the built-in interfacial potential. This facilitates a shift in the Fermi level, specifically within the topologically protected surface states that exist within the Bi_2_Se_3_ bulk band gap. The entire energy band diagram, including the CBM, the Dirac point of the surface states, and the valence band, shifts downward.

Although the propagating SPP mode is mainly confined to the Au/SiO_2_ interface, the TSS-related surface/interface states at the Au/Bi_2_Se_3_ contact can provide an additional bias-sensitive carrier modulation pathway. Under Fermi-level alignment and external bias, carrier redistribution through these surface/interface states perturbs the carrier density and effective dielectric boundary condition of the shared Au plasmonic layer, thereby increasing the voltage-dependent SPP attenuation and enhancing the ER.^17,35^

Therefore, the enhanced modulation is attributed to the combined effects of TSS-related interfacial carrier modulation, carrier-induced modification of the Au plasmonic layer, and possible bulk-related absorption/Burstein–Moss carrier-filling effects, rather than to a fully isolated TSS contribution.^18,34^

The novelty of this approach lies in the utilization of a 3D TI as an active material for plasmonic modulation. Unlike atomically thin 2D materials, the Bi_2_Se_3_ thin film provides a finite-thickness topological material platform with surface/interface carrier channels that can participate in electrically induced carrier redistribution at the Au/Bi_2_Se_3_ interface. Although the propagating SPP mode is mainly confined to the Au/SiO_2_ interface, the Bi_2_Se_3_-related interfacial carrier response can indirectly modify the effective dielectric boundary condition of the Au plasmonic layer and thereby affect the SPP attenuation. In addition, the topologically protected surface states of Bi_2_Se_3_ provide carrier-sensitive conducting channels at room temperature, enabling voltage-controlled carrier accumulation and redistribution near the Au/Bi_2_Se_3_ interface. Therefore, the Bi_2_Se_3_-integrated plasmonic waveguide can enhance the modulation depth through an additional interfacial carrier-modulation pathway rather than relying solely on the optical response of an atomically thin monolayer. Table 1 compares the modulation performance of the proposed device with representative visible-range plasmonic modulators incorporating 2D or layered materials. Since the reported modulation metrics and voltage definitions vary among different device configurations, the values are presented according to the definitions used in the original studies.^36–38^

**Table 1:** Comparison of Modulation Performance among Visible-Range Plasmonic Modulators Based on 2D and Layered Materials[Table-fn t1fn1]

Structure	Incident Light (λ)	Modulation metric	Bias Voltage	References
Graphene-Ag nanowire hybrid SPP	659 nm	0.08 dB/V^a^, 0.07 dB/μm	>25 V	^36^
Monolayer MoS_2_ + single Au nanodisk	650–685 nm	0.35 dB/V, 1.4 dB/μm^b^	0–8 V	^37^
Monolayer WS_2_–Ag slot plasmonic waveguide (simulation)	641 nm	13.58 dB/V, 22.75 dB/μm^c^	0.67 V	^38^
Ours (with Bi_2_Se_3_)	300–800 nm	0.42 dB/V, 0.0421 dB/μm	10 V	N/A

a–c Values in dB/V and dB/μm were derived from the ER, voltage, and device length reported in the original studies.

Compared with atomically thin 2D-material modulators, the proposed Bi_2_Se_3_-integrated plasmonic waveguide provides a broadband voltage-tunable response over a substantially wider spectral range. Although the required bias voltage is not the lowest among the listed devices, the finite thickness of the 3D TI film provides a carrier-sensitive material platform that can support surface/interface carrier redistribution at the Au/Bi_2_Se_3_ contact rather than relying on direct optical-field overlap with the Bi_2_Se_3_ layer. Meanwhile, the surface-state-related carrier channel of Bi_2_Se_3_ provides an additional carrier-sensitive pathway at the Au/Bi_2_Se_3_ interface, where bias-induced carrier accumulation and redistribution can modify the effective dielectric boundary condition of the shared Au plasmonic layer and thereby affect the attenuation of the Au/SiO_2_-supported SPP mode. This interfacial carrier modulation consequently affects the SPP transmission and enhances the overall modulation response. These features make the Bi_2_Se_3_ TI film a promising active material for hybrid plasmonic modulations.

In addition to its static modulation performance, evaluating the transient response and potential operating speed of the Bi_2_Se_3_-integrated plasmonic waveguide is critical for high-speed optical interconnect applications. Although the experimental 3-dB bandwidth was not directly measured in this work, the subwavelength confinement of the surface plasmon polariton (SPP) mode and the micrometer-scale interaction length are highly favorable for achieving compact, high-speed modulation. Given that the optical propagation time across a 100 μm-scale plasmonic channel is negligibly short, the practical frequency response is anticipated to be governed primarily by the electrical RC time constant of the electrode/waveguide architecture and the carrier redistribution dynamics at the Au/Bi_2_Se_3_ interface, rather than by optical transit limitations. To contextualize these dynamics, previously reported low-dimensional-material-based plasmonic modulators with micrometer-scale interaction lengths have demonstrated response times spanning the nanosecond to femtosecond regimes and bandwidths extending from the MHz to THz levels.^39,40^ Realizing this potential will require further strategic optimization of the electrode geometry, contact resistance, and device capacitance, alongside direct high-frequency S-parameter or time-resolved electro-optic measurements in future investigations to quantitatively determine the actual modulation bandwidth.

## Conclusions

In summary, we have demonstrated an actively tunable plasmonic waveguide modulator by integrating a transferred Bi_2_Se_3_ topological insulator thin film onto a prefabricated gold–dielectric waveguide platform. Adopting a polymer-assisted transfer technique enabled the incorporation of high-quality Bi_2_Se_3_ films, effectively overcoming the challenges associated with direct growth on nanostructured substrates. Following Bi_2_Se_3_ integration, the transmission spectrum exhibits an additional apparent peak displacement of up to 49.2 nm compared with the reference device. This displacement is attributed to the combined effects of Bi_2_Se_3_-induced interfacial carrier redistribution, carrier-induced modification of the shared Au plasmonic layer, possible Burstein–Moss-related absorption modification, and accumulated wavelength-dependent propagation loss, rather than direct evanescent-field interaction with the Bi_2_Se_3_ film. In addition, electrical biasing results in an enhanced ER of up to 4.21 dB. These modulation effects arise mainly from additional interfacial carrier dynamics at the Au/Bi_2_Se_3_ interface, which perturb the carrier distribution and effective dielectric boundary condition of the shared Au plasmonic layer, together with possible Bi_2_Se_3_-related Burstein–Moss carrier-filling effects. This study highlights the viability of topological insulator-based hybrid plasmonic waveguides for developing compact, broadband, and energy-efficient electro-optic modulators, offering promising prospects for next-generation integrated nanophotonic systems.

## Supplementary Material



## Data Availability

The data sets generated during or analyzed during the current study are available from the corresponding author on reasonable request.

## Notes

Additional experimental results and videos supporting the main text. Figure S1 shows the effect of KOH concentration on the transfer quality of Bi_2_Se_3_ thin films, highlighting the trade-off between transfer area and film uniformity. Figure S2 compares the ERs of a 100 μm-long waveguide of light incidence in the TE and TM polarizations. Figure S3 presents the normalized transmission spectra of Au/SiO_2_/Si_3_N_4_ waveguides with different lengths, without and with Bi_2_Se_3_. These data help illustrate the influence of waveguide length and the role of Bi_2_Se_3_ in optical modulation (PDF)

## Abbreviations

SPPs: surface plasmon polaritons
ER: extinction ratio
MIM: metal–insulator–metal
IMI: insulator–metal–insulator
TI: topological insulator
CBM: conduction band minimum
EF: Fermi level
RIE: reactive ion etching
PMMA: poly­(methyl methacrylate)
