# Supplementary material for: Plasmonic Waveguide Modulator Integrated with a Transferred Bi2Se3 Topological Insulator Film
Source: ACS Omega. 2026 Aug 3;11(32):48334–43. doi: 10.1021/acsomega.6c04951 (PMC13491947; doi:10.1021/acsomega.6c04951)
Supplement: Supplementary file 1 [file ao6c04951_si_001.pdf]

Supporting Information of A Plasmonic Waveguide Modulator Integrated with a Transferred Bi_2Se_3 Topological Insulator Film

Cheng-Yi Cheng[1], Kuan-Han Wu[1], Jiang-Qin Shi[1], Rohit Gupta[1], Jian-Jang Huang[1,2,]*

[1]Graduate Institute of Photonics and Optoelectronics, National Taiwan University, No. 1, Sec. 4, Roosevelt Rd., Taipei 10639, Taiwan

[2]Department of Electrical Engineering, National Taiwan University, No. 1, Sec. 4, Roosevelt Rd., Taipei 10639, Taiwan

*jjhuang@ntu.edu.tw

CONTENTS

Figure S1. Effect of KOH concentration on the transfer quality of Bi_2Se_3 thin films.

Figure S2. Comparison of the ERs for a 100 μm-long Waveguide Under TE and TM Polarization Light Incidence (a) TM-polarized and (b) TE-polarized incident light. (c) Comparisons of the ERs under TE and TM polarized light incidence at various bias voltages.

Figure S3. Normalized transmission spectra of $Au/SiO_2/Si_3N_4$ waveguides with different lengths. The spectra without the Bi_2Se_3 film are shown in dashed lines, while those with the Bi_2Se_3 are in solid lines.

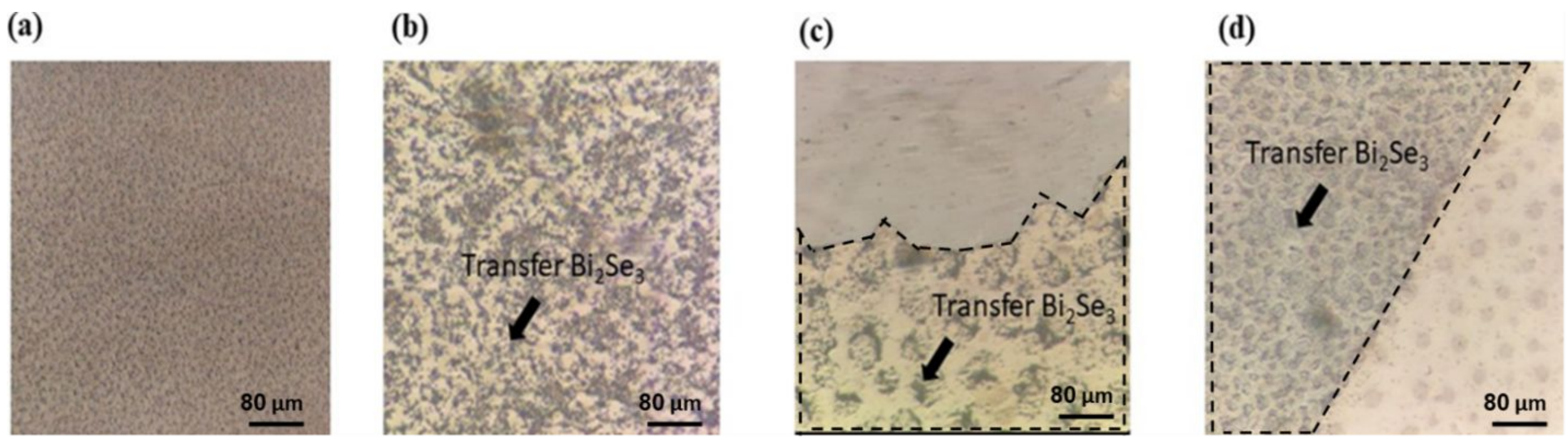

Figure S1. Effect of KOH concentration on the transfer quality of Bi_2Se_3 thin films. **(a)** As-deposited Bi_2Se_3 film prepared by chemical vapor deposition. **(b-d)** Transfer processes were performed with KOH volumes of 40 mL (21.75 wt.), 60 mL (17.80 wt.), and 80 mL (13.05%wt.), respectively, while maintaining a constant water volume of 160 mL.

Fig. S1 demonstrates the effect of KOH concentration on the transfer quality of Bi_2Se_3 thin films. Reducing the KOH volume from 80 mL to 40 mL increases the transferred area of Bi_2Se_3, albeit at the expense of a longer etching duration. However, despite the larger transfer area observed at 40 mL and 60 mL of KOH, the overall transfer quality significantly deteriorates compared to that obtained using 80 mL of KOH. Furthermore, low KOH concentration not only compromises the uniformity of the transferred film but also leads to residual photoresist patches that cannot be fully removed, thereby affecting the surface integrity.

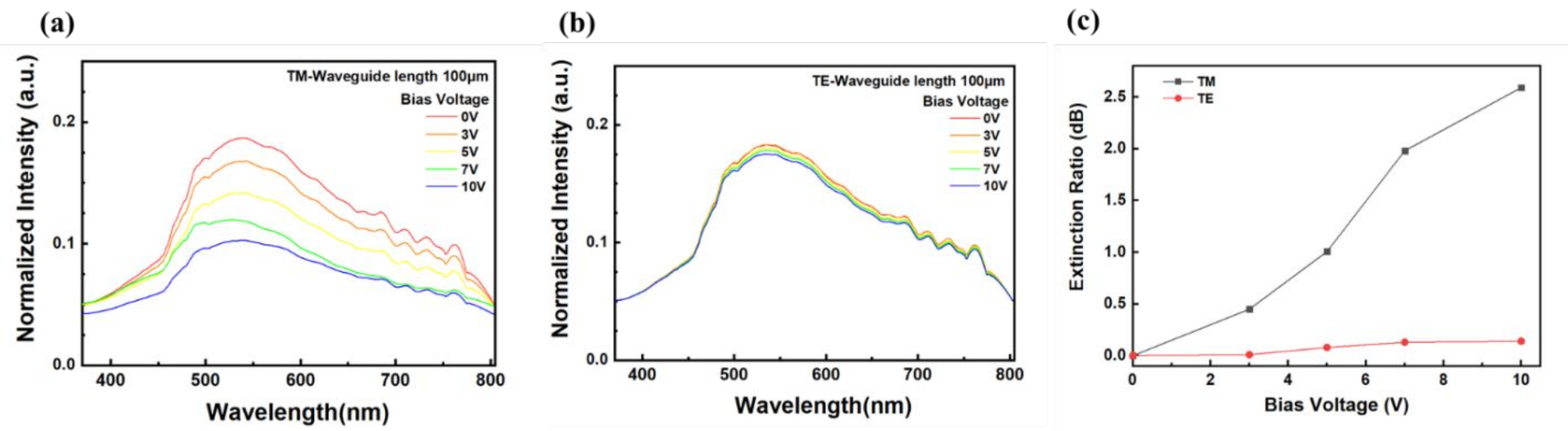

Figure S2. Comparison of the ERs for a 100 μm-long Waveguide under TE and TM polarized light incidence. (a) TM-polarized and (b) TE-polarized incident light. (c) Comparisons of the ERs under TE and TM polarized light incidence at various bias voltages.

Fig. S2 presents the normalized transmission spectra of a 100-μm-long plasmonic waveguide under different bias voltages for (a) TM-polarized and (b) TE-polarized incident light. In subfigure a, a clear voltage-dependent modulation is observed for TM polarization. In contrast, subfigure b shows almost no modulation under TE-polarized excitation. This difference arises because only TM-polarized light can excite surface plasmon polaritons (SPPs), leading to a bias-dependent change in transmission. Fig. S2(c) further compares the ERs for the two polarization states. Consistent with the spectra, the ER varies with bias only for TM-polarized light, while TE-polarized light exhibits negligible change.

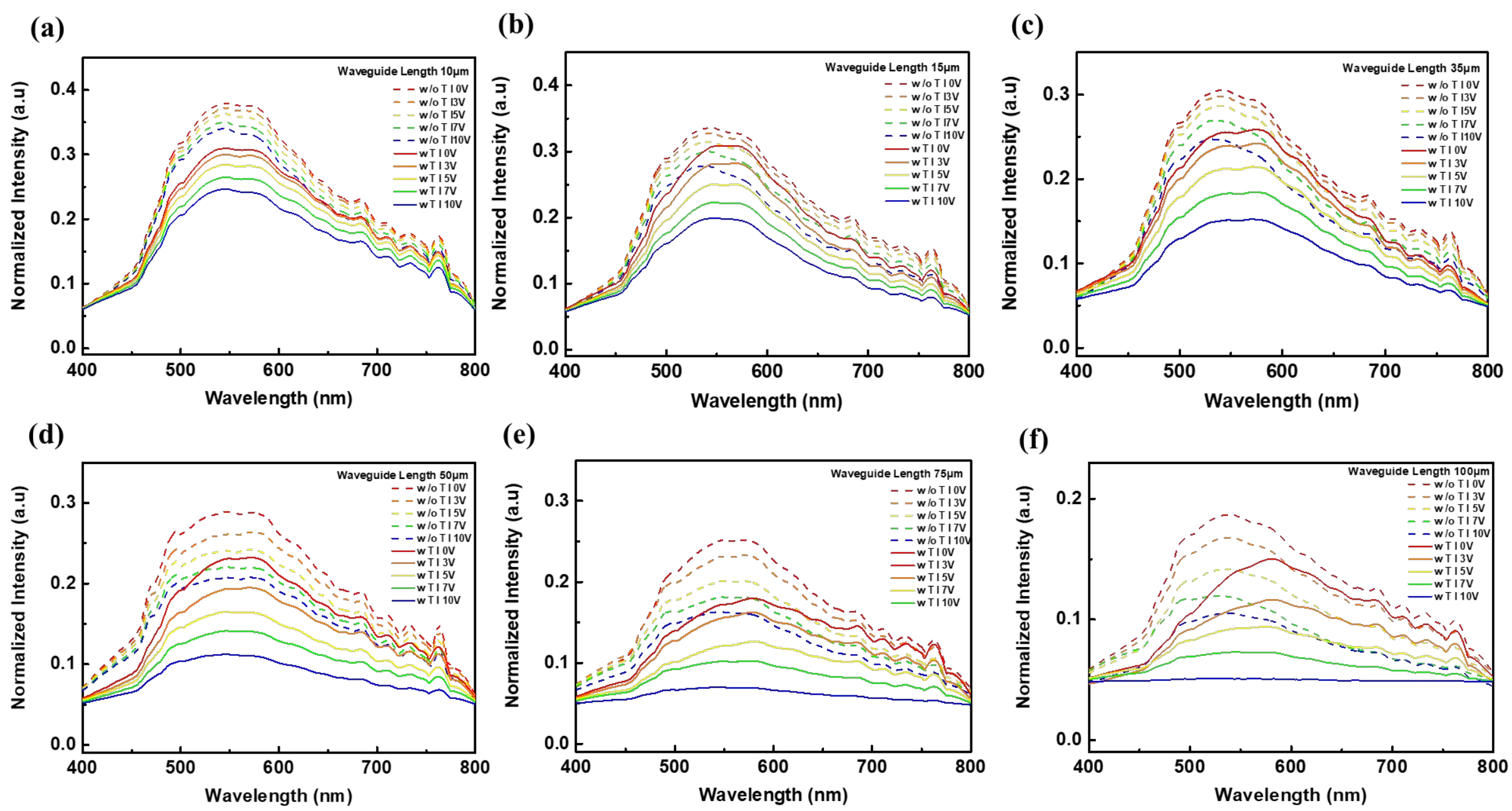

Figure S3. Normalized transmission spectra of $Au/SiO_2/Si_3N_4$ waveguides (with and without Bi_2Se_3) with length of **(a)** 10, **(b)** 15, **(c)** 35, **(d)** 50, **(e)** 75, **(f)** 100 μm.

Fig. S3 shows the normalized transmission spectra of $Au/SiO_2/Si_3N_4$ waveguides with and without a Bi_2Se_3 thin film, measured for different lengths under various applied bias voltages. The spectra in subfigures a–f correspond to waveguide lengths of 10, 15, 35, 50, 75, and 100 μm, respectively. For each length, measurements were carried out as the bias voltage was varied from 0 V to 10 V, both prior to and following Bi_2Se_3 deposition (denoted as "w/o TI" and "w/ TI", respectively). Incorporating the Bi_2Se_3 layer leads to a marked enhancement in the bias-dependent modulation response, as evidenced by the greater reduction in transmission intensity relative to uncoated devices. This effect becomes increasingly prominent with longer propagation lengths, indicating the cumulative influence of the Bi_2Se_3-induced modulation along the waveguide.

AUTHOR INFORMATION

Corresponding Author

*E-mail: jjhuang@ntu.edu.tw
